# Novel Insights Into the Neurobiology of the Antidepressant Response From Ketamine Research: A Mini Review

**DOI:** 10.3389/fnbeh.2021.759466

**Published:** 2021-12-03

**Authors:** Michael Colla, Hanne Scheerer, Steffi Weidt, Erich Seifritz, Golo Kronenberg

**Affiliations:** Department of Psychiatry, Psychotherapy and Psychosomatics, Hospital of Psychiatry, University of Zurich, Zurich, Switzerland

**Keywords:** depression, antidepressant, treatment-resistant depression, ketamine, BDNF, neurogenesis, monoamines, glutamate

## Abstract

The serendipitous discovery of ketamine’s antidepressant effects represents one of the major landmarks in neuropsychopharmacological research of the last 50 years. Ketamine provides an exciting challenge to traditional concepts of antidepressant drug therapy, producing rapid antidepressant effects seemingly without targeting monoaminergic pathways in the conventional way. In consequence, the advent of ketamine has spawned a plethora of neurobiological research into its putative mechanisms. Here, we provide a brief overview of current theories of antidepressant drug action including monoaminergic signaling, disinhibition of glutamatergic neurotransmission, neurotrophic and neuroplastic effects, and how these might relate to ketamine. Given that research into ketamine has not yet yielded new therapies beyond ketamine itself, current knowledge gaps and limitations of available studies are also discussed.

## Introduction

Ketamine, synthesized in 1962 by the research team of Calvin Stevens, was the culmination of Parke-Davis’s drive to find a short-acting intravenous anesthetic with favorable cardiovascular and respiratory characteristics (Mion, 2017). Ketamine replaced its congener phencyclidine (PCP), which, after a brief period of use as an anesthetic agent under the brand name Sernyl ^®^, had to be abandoned owing to high rates of postoperative dysphoria and hallucinations (Meyer et al., 1959). The distinctive state produced by ketamine — characterized by analgesia, catalepsy, and amnesia, while maintaining respiratory reflexes and hemodynamic stability — was first described by Corssen and Domino (1965), who dubbed it “dissociative anesthesia.” Its wide therapeutic index makes ketamine an excellent agent for use in emergency medical practice, battlefield pain management, and, more generally, in resource-stripped settings such as the developing world. Ketamine remains on the most recent WHO Model List of Essential Medicines as an injectable general anesthetic (World Health Organization [WHO], 2019).

Ketamine burst on the scene of antidepressant psychopharmacology in 2000 with the publication of its first double-blind placebo-controlled trial in major depression. This pilot investigation of seven patients found significant improvements in mood within 72 h of a single subanesthetic dose of intravenous racemic ketamine hydrochloride (Berman et al., 2000). A number of follow-up studies have confirmed the fast-onset antidepressant effects of ketamine infusions (Zarate et al., 2006a; aan het Rot et al., 2010; Murrough et al., 2013a,b). Moreover, adjunctive intravenous ketamine has emerged as a powerful new treatment option for patients suffering from treatment-resistant depression (TRD; Diazgranados et al., 2010; Fava et al., 2020). In the interim, variant forms of ketamine therapy including treatment with the S-enantiomer (i.e., S-ketamine) and administration via the nasal (Popova et al., 2019) and oral route (Domany et al., 2019) have also been demonstrated to confer rapid antidepressant benefit. Table 1 summarizes key studies of ketamine in depression.

**TABLE 1:** Overview of key studies of ketamine in depression.

Number of patients investigated	Study design	Route of administration	Patient characteristics	Results	References
9 (2 drop-outs)	Randomized, double-blind study of single dose of ketamine hydrochloride (0.5 mg/kg); two treatment days, at least 1 week apart	Intravenous	Recurrent unipolar depression and bipolar depression; unmedicated patients	Significant improvement within 72 h after ketamine (HDRS)	Berman et al., 2000
18 (1 drop-out)	Randomized, placebo-controlled, double-blind crossover study of single dose of ketamine hydrochloride (0.5 mg/kg)	Intravenous	Major depressive disorder, recurrent, without psychotic features; unmedicated patients	Significant improvement within 110 min after ketamine which remained significant throughout the following week (HDRS)	Zarate et al., 2006a
10	Repeated-dose open-label ketamine hydrochloride (0.5 mg/kg; six infusions over 12 days)	Intravenous	Medication free symptomatic patients suffering from treatment-resistant depression (patients excluded if they had lifetime history of psychotic symptoms or hypomania/mania)	The mean (SD) reduction in MADRS scores after sixth infusion was 85% (12%).	aan het Rot et al., 2010
73	Two-site, parallel-arm, randomized controlled trial of a single dose of ketamine hydrochloride (0.5 mg/kg) compared to active placebo (i.e., midazolam, 0.045 mg/kg) in a 2:1 ratio.	Intravenous	Treatment-resistant major depression (patients excluded if they had lifetime history of psychotic symptoms or bipolar disorder); unmedicated patients (with the exception of a stable dose of a non-benzodiazepine hypnotic).	Ketamine group showed greater improvement (MADRS score) than midazolam group 24 h after treatment	Murrough et al., 2013a
24	Series of up to six infusions of ketamine hydrochloride (0.5 mg/kg) administered open-label three times weekly over a 12-day period.	Intravenous	Treatment-resistant major depression (patients excluded if they had lifetime history of psychotic symptoms or bipolar disorder); patients free of antidepressant medication during infusion period	Large mean decrease in MADRS score at 2 h after first ketamine infusion which was largely sustained for the duration of the infusion period.	Murrough et al., 2013b
18	Randomized, placebo-controlled, double-blind, crossover, add-on study of ketamine hydrochloride (0.5 mg/kg) or placebo combined with lithium or valproate therapy on 2 test days 2 weeks apart	Intravenous	Treatment resistant bipolar I or II depression without psychotic features	Depressive symptoms significantly improved within 40 min in subjects receiving ketamine compared with placebo; improvement remained significant through day 3.	Diazgranados et al., 2010
99	Double-blind ketamine or placebo added to ongoing antidepressant therapy; patients randomly assigned to one of five study arms in a 1:1:1:1:1 fashion: single dose of ketamine 0.1 mg/kg (*n* = 18), 0.2 mg/kg (*n* = 20), 0.5 mg/kg (*n* = 22), 1.0 mg/kg (*n* = 20), and a single dose of midazolam 0.045 mg/kg (*n* = 19)	Intravenous	Treatment-resistant MDD (patients excluded if they had history of bipolar disorder, schizophrenia, or schizoaffective disorders, or any history of psychotic symptoms in current or previous depressive episodes)	Evidence for the efficacy of the 0.5 mg/kg and 1.0 mg/kg subanesthetic doses of IV ketamine, no clear or consistent evidence for clinically meaningful efficacy of lower doses	Fava et al., 2020
197 patients completed 28-day double-blind treatment phase.	Phase 3, double-blind, active-controlled, multicenter study of esketamine (56 and 84 mg versus placebo)	Intranasal	Treatment resistant moderate to severe MDD (key exclusion criteria: diagnosis of psychotic disorder, major depressive disorder with psychotic features, bipolar or related disorders, borderline, antisocial, histrionic, or narcissistic personality disorder)	Change in MADRS score with esketamine plus antidepressant significantly greater than with antidepressant plus placebo at day 28, clinically meaningful improvement observed in the esketamine plus antidepressant arm at earlier time points	Popova et al., 2019
41	Randomized, double-blind, placebo-controlled, proof-of-concept trial; participants received either 1 mg/kg oral ketamine or placebo thrice weekly for 21 days	Oral	Treatment-resistant MDD (key exclusion criteria: psychotic disorder or psychotic symptoms, bipolar disorder)	Reduction in MADRS score on day 21 significantly greater in the ketamine group than in the control group. Six participants in ketamine group (27.3%) achieved remission compared with none of the controls.	Domany et al., 2019

From a neuroscience perspective, the uncanny rapidity of ketamine’s antidepressant action (often within a few hours) sets it apart from conventional antidepressants, providing a new window on the neurobiology of the antidepressant response with exciting possibilities for translational and, maybe even more interesting, reverse translational research. The purpose of this mini-review is, therefore, to provide an overview of current thinking on ketamine’s putative mechanisms of action within the context of antidepressant drug discovery and development.

## Monoamine Mechanisms

The short history of the development of antidepressant drugs is riddled with accidental yet transformative discoveries. At the risk of recounting well-known facts, here is a summary of the milestones: Iproniazid, initially developed and marketed by Hoffmann La-Roche as an antibiotic to treat tuberculosis (Marsilid ^®^), was serendipitously identified as possessing antidepressant characteristics (Loomer et al., 1957; deVerteuil and Lehmann, 1958). A connection was quickly made with iproniazid’s strong inhibitory effect on monoamine oxidase (MAO), paving the way for the targeted development of other, more refined, and ultimately safer MAO inhibitors, which are still widely prescribed today (e.g., Stefanis et al., 1982). The antidepressant activity of imipramine, the first tricyclic antidepressant, was recognized almost coevally with the discovery of iproniazid’s antidepressant properties (Kuhn, 1957). Inhibition of the reuptake of biogenic amines was swiftly identified as the primary molecular mechanism of tricyclics (Axelrod et al., 1961; Carlsson et al., 1966, 1968; Fuxe and Ungerstedt, 1968). The observation that blood-pressure lowering drug reserpine may precipitate depression (Harris, 1957) provided further support for a link between brain levels of biogenic amines and mood states. Taken together, and in historical perspective, this “monoamine hypothesis” of depression has proven incredibly useful in the development of newer classes of antidepressants (such as selective serotonin reuptake inhibitors, noradrenaline reuptake inhibitors, dual reuptake inhibitors, etc.) that are usually superior to the older compounds with comparable efficacy yet fewer side effects and a greater therapeutic index. Nevertheless, the clinical limitations of monoamine-based agents, in particular relatively high rates of non-response and even resistance to treatment, have long led to calls to focus more research on alternative mechanisms (Berton and Nestler, 2006).

An obvious conceptual problem with the monoamine hypothesis lies in the fact that changes in neurotransmitter concentrations (along with the onset of typical side effects) occur within a few hours while conventional antidepressants typically require several days to weeks to take effect. Neurobiological research into the mechanisms underpinning the antidepressant response has therefore pivoted to longer-term adaptive changes downstream of the acute effects on biogenic amines.

Ketamine’s principal pharmacological action is as an *N*-methyl-D-aspartate (NMDA) receptor antagonist. However, in a manner somewhat reminiscent of clozapine, ketamine is a “dirty” drug. Multiple off-target effects including on monoamine systems need to be considered. *In vitro*, ketamine displays affinity to dopamine D2 and serotonin 5-HT2 receptors in the same range as its affinity for the NMDA receptor (Kapur and Seeman, 2002). It has also been reported that ketamine inhibits monoamine transporters in cultured cells (Nishimura et al., 1998) and blocks the uptake of [3H]-dopamine into rat striatal synaptosomes (Keita et al., 1996).

Repeated ketamine injections increase the firing rate of norepinephrine neurons in the locus coeruleus and of dopaminergic neurons in the ventral tegmental area in rats (Iro et al., 2021). Microdialysis studies have demonstrated increased serotonin release by ketamine in the rodent prefrontal cortex (Ago et al., 2019; López-Gil et al., 2019). Several groups have found that serotonin depletion abrogates the antidepressive-like effects of ketamine in the forced swim test (Gigliucci et al., 2013; Fukumoto et al., 2015; du Jardin et al., 2016; Pham et al., 2017). Even so, measurable occupancy of the serotonin transporter *in vivo* was not detectable by positron emission tomography in twelve healthy human subjects after infusion of an antidepressant dose of ketamine (Spies et al., 2018).

While the available literature indicates that ketamine leads to increased dopamine levels in frontal cortex, striatum, and nucleus accumbens in rodents, the picture is less clear for the primate and human brain, given methodological issues and the scant available literature (Kokkinou et al., 2018). From a clinical perspective, the fact that haloperidol is able to ameliorate ketamine-induced psychosis argues for a role of dopaminergic pathways in ketamine’s psychotropic effects (Giannini et al., 2000).

## Ketamine and the Glutamatergic System

**FIGURE 1:**
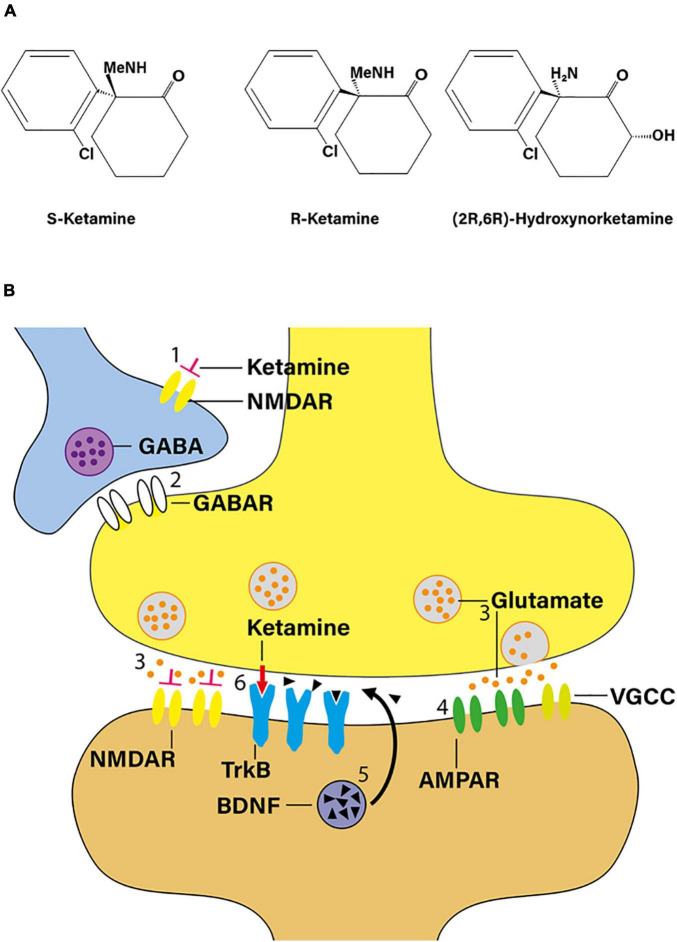
Ketamine as a novel antidepressant. **(A)** Structural formula of S-ketamine, R-ketamine, and R-ketamine metabolite 2R,6R-hydroxynorketamine (adapted from Zanos et al., 2016). Me, methyl moiety. **(B)** According to the “disinhibition hypothesis” of ketamine action, NMDA receptor blockade by ketamine may increase glutamatergic outflow. When administered in a subanesthetic dose, ketamine blocks NMDA receptors on γ-aminobutyric acid (GABA) interneurons (1), thereby reducing GABA release (2) on principal neurons, and, in turn, increasing presynaptic release of glutamate (3). Preferential activation of postsynaptic α-amino-3-hydroxy-5-methyl-4-isoxazole-propionic acid receptors (AMPAR) in mood-regulating synapses (4) is believed to play a critical role in mediating ketamine’s rapid antidepressant response, triggering downstream changes such as inducing BDNF signaling (5). In addition, ketamine may also interact with the TrkB receptor directly (red arrow; 6). TrkB, tropomyosin receptor kinase B; VGCC, voltage-gated calcium channel (adapted from Shinohara et al., 2020).

Racemic ketamine acts as a non-competitive NMDA receptor antagonist (Figure 1A). It is believed that the dissociative and psychotomimetic effects of PCP and ketamine relate directly to the affinity of these molecules to the NMDA receptor. Based on displacement binding studies with [3H]-MK801 as the marker ligand, S-ketamine exhibits an approximately three- to fourfold higher affinity to the NMDA receptor than R-ketamine (Moaddel et al., 2013). The pharmacokinetic profiles of racemic ketamine and its two enantiomers do not differ significantly in humans (White et al., 1985). Serum ketamine concentrations at the point of regaining consciousness and orientation during the course of experimental anesthesia of human volunteers indicate an S:R ketamine isomer potency ratio of 4:1. Similarly, S-ketamine has an approximately three- to fivefold greater ability to impair psychomotor function than R-ketamine (White et al., 1985). The available literature, though scant, seems to suggests that, in humans, subanesthetic doses of R-ketamine lack the dissociative potential of racemic ketamine (Vollenweider et al., 1997; Leal et al., 2021).

It is tempting to speculate that the dissociative and the antidepressant effects of ketamine might be separable. In the context of double-blind placebo-controlled drug testing, this is a complex issue because questions around functional unblinding due to ketamine’s dissociative effects and the potential use of active comparators have to be considered (Ballard and Zarate, 2020). At least so far, the bulk of the available clinical evidence seems to favor an association between racemic ketamine’s hallucinogenic/dissociative and antidepressant effects (Mathai et al., 2020).

While clinical research into a possible role for R-ketamine in depression is still in its infancy, sufficient data has already accrued to recommend the use of S-ketamine. Intravenous S-ketamine has been shown to produce rapid onset of robust antidepressant effects in patients with TRD after a 40-min infusion (Singh et al., 2016). Further, there is meta-analytical evidence for the adjunctive intranasal use of S-ketamine in TRD and in depressed patients with acute suicidality (Papakostas et al., 2020). Moreover, a recent randomized double-blind head-to-head comparison of intravenous S-ketamine (0.25 mg/kg) and racemic ketamine (0.5 mg/kg) as adjunctive therapy in TRD confirmed non-inferiority of S-ketamine (Correia-Melo et al., 2020).

*N*-methyl-D-aspartate receptor blockade may augment glutamatergic outflow, e.g., in the prefrontal cortex. Indeed, one plausible mechanism of this seemingly paradoxical effect is that ketamine, when administered in a subanesthetic dose, blocks NMDA receptors on γ-aminobutyric acid interneurons, thereby increasing presynaptic release of glutamate (Moghaddam et al., 1997; Pothula et al., 2020). According to this “disinhibition hypothesis” (Figure 1B), downstream activation of α-amino-3-hydroxy-5-methyl-4-isoxazole-propionic acid (AMPA) receptors in mood-regulating synapses is believed to play a crucial role in mediating ketamine’s rapid antidepressant response as evidenced by the fact that pre-treatment with NBQX, an AMPA receptor antagonist, attenuates the behavioral effects of ketamine in experimental mice and rats (Maeng et al., 2008; Koike and Chaki, 2014). It thus appears that, ultimately, ketamine produces increased glutamatergic throughput of AMPA receptors, as compared to NMDA receptors, triggering rapid downstream changes on the molecular, structural, and network levels (Figure 1B; Jourdi et al., 2009; Li et al., 2010; Autry et al., 2011).

While clinical research has, so far, focused primarily on the S-ketamine stereoisomer, it has been hypothesized, based on behavioral studies in experimental mice, that R-ketamine should show the greater antidepressant potency (Yang et al., 2015; Zanos et al., 2016). To our knowledge, there is currently only one small pilot trial that has investigated the effects of R-ketamine in major depression (Leal et al., 2021). That open-label study of seven patients reported a significant decrease in Montgomery-Åsberg Depression Rating Scale scores within 24 h of a single intravenous infusion of R-ketamine (0.5 mg/kg).

There is extensive metabolism of ketamine stereoisomers via cytochrome P450 enzymes producing a broad array of catabolites including norketamine, hydroxyketamines, dehydronorketamine, and the hydroxynorketamines (Kharasch and Labroo, 1992; Desta et al., 2012). In particular, potent antidepressant properties have been ascribed to the (2R,6R)-hydroxynorketamine [(2R,6R)-HNK] metabolite (Figure 1A), which is exclusively derived from R-ketamine (Zanos et al., 2016). Mechanistically, (2R,6R)-HNK acts through AMPA receptor-mediated mechanisms, with the AMPA receptor antagonist NBQX reversing its antidepressant-like effects (Zanos et al., 2016). Moreover, (2R,6R)-HNK recapitulates key downstream events observed in the rodent brain in response to ketamine such as increased neurotrophic signaling and rapid dendritic and synaptic plasticity (Autry et al., 2011; Zanos et al., 2016).

## Neurotrophic Signaling, Neuroplasticity, and Stress

Profound structural changes such as neuronal atrophy, loss of synapses, and a decrease in hippocampal neurogenesis reflect the deleterious effects of stress, stress hormones, and major depression on the brain (Duman et al., 2016). Brain-derived neurotrophic factor (BDNF)/tropomyosin receptor kinase B (TrkB) signaling is of crucial importance to neuronal plasticity, morphogenesis, and survival (Huang and Reichardt, 2001). Numerous pre-clinical studies have connected stress and an excess of corticosteroids with reduced BDNF signaling in depression-related brain areas (Smith et al., 1995; Schaaf et al., 2000; Vollmayr et al., 2000; Oh et al., 2019). Conversely, bilateral infusion of BDNF into the hippocampal dentate gyrus has been shown to produce antidepressant-like effects in behavioral models of depression (Shirayama et al., 2002). Antidepressant interventions such as electroconvulsive therapy (Nibuya et al., 1995), physical activity (Sleiman et al., 2016), and conventional antidepressant pharmacotherapy (Conti et al., 2002) have all been linked with a rise in brain BDNF levels. Likewise, ketamine administration has been shown to raise BDNF mRNA and protein levels in hippocampus (Choi et al., 2017). BDNF signaling seems to be central to ketamine’s distinct antidepressant activity because ketamine fails to produce rapid antidepressant-like effects in either BDNF or TrkB conditional knockout mice (Autry et al., 2011). Quite unexpectedly, some very recent research has demonstrated an exciting new mode of action of several antidepressants, including ketamine, beyond increasing BDNF concentrations, namely, to directly bind to TrkB (Casarotto et al., 2021). Antidepressant binding to TrkB could then facilitate BDNF action and the attendant cellular as well as structural plasticity (Casarotto et al., 2021). An important intracellular signaling pathway activated in response to ketamine is the mammalian target of rapamycin pathway. Activation of this pathway promotes rapid synaptic plasticicity with increased synaptic signaling proteins and increased number and function of synapses (Li et al., 2010). In this context, and given that the anti-dementia drug memantine, which shares with ketamine the property of non-competitive NMDA antagonism, is widely prescribed in Alzheimer’s disease, it may be worthwhile to assess the effects of ketamine in patients with dementia (Smalheiser, 2019).

So far, few studies have investigated the effects of ketamine on hippocampal neurogenesis (Deyama and Duman, 2020). It has been reported that ketamine increases cell proliferation in the hippocampal dentate gyrus of rats showing a depressive-like phenotype (Michaëlsson et al., 2019). However, since neurogenesis is a multi-step process that unfolds over several weeks (Kempermann et al., 2004), it is unlikely that an overall increase in neurogenesis explains ketamine’s rapid antidepressant effects. Still, increased recruitment of adult-born neurons into hippocampal circuitry (i.e., an acceleration in the final stages of neurogenesis) in response to ketamine is an obvious possibility, especially considering the importance of these immature cells for shaping memory processes (Anacker and Hen, 2017).

## Open Questions and Outlook

An honest appraisal of where the field stands today must acknowledge the fact that, so far, “decades of ‘murinization”’ have contributed relatively little to antidepressant development (Holsboer, 2014). From the sole perspective of drug discovery, the poor predictability of antidepressant efficacy based on behavioral assays in rodents is probably chief among today’s challenges. On the other hand, it should be noted that the concept of NMDA antagonism in the treatment of depression was developed against a rich backdrop of experimental research (reviewed in Skolnick et al., 1996), demonstrating, among other things, that chronic administration of desipramine inhibits glutamatergic neurotransmission at NMDA receptors (Mjellem et al., 1993), and that both conventional antidepressants and electroconvulsive therapy alter the ligand-binding properties of the NMDA receptor complex (Paul et al., 1993, 1994). Given the short nature of this mini-review, the considerable body of preclinical evidence demonstrating ketamine’s antidepressant activity in rodent models of depression has been largely passed over. For a detailed overview of this subject, the reader is referred to Polis et al. (2019) and Rincón-Cortés and Grace (2020).

How will the field evolve in the future? As a logical next step, the R-ketamine enantiomer is currently in the early stages of clinical development. Moreover, certain ketamine metabolites may hold promise as possessing equal antidepressant efficacy to the racemic parent molecule, possibly with fewer side effects, especially (2R,6R)-HNK. From a broader view, however, the prospect of discovering other molecules, not directly related to ketamine itself but tapping into the same neurobiological mechanisms, remains uncertain, at least for the time being. So far, the principle of NMDA antagonism has, unfortunately, not translated into tangible new drugs. Also, side-effects beyond psychotic symptoms have to be considered. Merck & Co’s dizocilpine (commonly referred to as MK-801 in the lab), a strong NMDA receptor antagonist, was shown to produce acute pathomorphological lesions in specific populations of neurons when administered acutely to adult rats in comparatively low doses (Olney et al., 1989). MK-801 is no longer in active clinical development for this reason. Similar evidence of neurotoxicity (the eponymous “Olney’s” lesions) has also been observed in experimental rodents after ketamine and PCP (reviewed in Ellison, 1995) and, more worryingly, in human ketamine addicts (Wang et al., 2013). Given these findings, it will be important, from a safety standpoint, to monitor the long-term effects of NMDA antagonist therapy (including with ketamine and S-ketamine) on brain structure and patients’ cognitive trajectories.

Moving beyond neurotoxicity, which may represent a class effect, investigations of NMDA receptor antagonists other than ketamine in depression have, so far, failed to produce clinically relevant outcomes. Memantine proved ineffective as an antidepressant in two double-blind placebo-controlled trials (Zarate et al., 2006b; Smith et al., 2013). Similarly, rislenemdaz (also known as MK-0657), an NR2B subunit-specific NMDA receptor antagonist, failed to produce antidepressant effects in TRD, either when used as a monotherapy or in conjunction with other antidepressants (Ibrahim et al., 2012; Henter et al., 2021). Lanicemine, an NMDA blocker with low rates of associated psychotomimetic effects, does not come near to replicating ketamine’s antidepressant effects (Zarate et al., 2013; Sanacora et al., 2017). More recently, three phase-III clinical trials of rapastinel, an NMDA receptor modulator with glycine-site partial agonist features, also failed to demonstrate antidepressant effects (Henter et al., 2021). This outcome is sobering, given that pre-clinical research had demonstrated antidepressant-like effects of rapastinel in mice and rats (Burgdorf et al., 2013; Yang et al., 2016).

The possibility of still other modes of action should also not be overlooked. It has long been known that ketamine possesses certain anti-inflammatory properties, which may especially benefit patients undergoing major surgery or septic patients requiring sedation (Kawasaki et al., 1999; Welters et al., 2011). Intriguingly, lipopolysaccharide-induced sickness behavior in mice can be blocked by ketamine (Walker et al., 2013). Moreover, the antidepressant effects of the two ketamine enantiomers in the chronic social defeat stress model of depression have been linked with restoration of gut microbiota in mice (Yang et al., 2017).

Opioid effects have also been implicated in ketamine’s clinical profile. Both S- and R-ketamine bind to and activate mu and kappa opioid receptors (Bonaventura et al., 2021). Further, it has recently been reported that naltrexone blocks the antidepressant effects of ketamine in depressed patients (Williams et al., 2018).

In the aggregate, ketamine represents the first major breakthrough in antidepressant development in the last half-century. As described above, it engages novel mechanisms beyond monoaminergic neurotransmission, resulting in a much faster onset of action than conventional monoamine-based therapeutics. Although much remains to be elucidated, the advent of ketamine signals exciting new opportunities to extend and refine our knowledge of the neurobiological mechanisms underlying the antidepressant response. Given the accruing evidence of ketamine’s therapeutic effects in TRD, it seems that the time has arrived to assign a central position to ketamine as an augmentation in the treatment algorithms for TRD patients.

## Author Contributions

GK drafted the manuscript with substantive input from all authors. All authors contributed to the article and approved the submitted version.

## Conflict of Interest

The authors are conducting a study of oral ketamine in TRD funded by Ketabon GmbH. ES serves on advisory boards of Janssen.

## Publisher’s Note

All claims expressed in this article are solely those of the authors and do not necessarily represent those of their affiliated organizations, or those of the publisher, the editors and the reviewers. Any product that may be evaluated in this article, or claim that may be made by its manufacturer, is not guaranteed or endorsed by the publisher.
